# Using probiotic supplementation to support bone health in postmenopausal women: a randomized, double-blind, parallel, placebo-controlled, multi-center study

**DOI:** 10.1007/s11657-025-01589-2

**Published:** 2025-07-27

**Authors:** Jenalyn L. Yumol, Sylvie Binda, Varuni Nagulesapillai, Renu Bhardwaj, Wendy E. Ward

**Affiliations:** 1 Department of Kinesiology, Brock University, St. Catharines, ON Canada; 2 Centre for Bone and Muscle Health, Brock University, 1812 Sir Isaac Brock Way, St. Catharines, ON L2S3A1 Canada; 3 Rosell Institute for Microbiome and Probiotics, Montreal, QC Canada

**Keywords:** Probiotic, Postmenopausal, Bone mineral density, Fracture, Bone turnover markers

## Abstract

***Summary*:**

Studies using rodent models have demonstrated the ability for probiotics to attenuate estrogen-related bone loss, but findings in humans are limited. Postmenopausal women consuming a novel combination of bacteria strains as a probiotic supplement demonstrated no changes in bone health outcomes.

**Purpose:**

This study determined if a probiotic supplement could attenuate the loss of femoral neck bone mineral density (BMD) and assessed its effect on fracture risk and markers of bone cell activity.

**Methods:**

Seventy-two postmenopausal women (40–59 years) were randomized to a daily probiotic supplement or placebo for 48 weeks. Femoral neck BMD was assessed at weeks 0 and 48 using DXA along with fracture risk using the FRAX® assessment tool. Serum procollagen type 1 N-terminal propeptide (P1NP), bone-specific alkaline phosphatase (BALP), cross-linked C-telopeptide of type I collagen (CTx), and osteocalcin (OC) were analyzed at weeks 0, 12, 24, and 48.

**Results:**

There was no significant time by treatment interaction (*p* > 0.05) for femoral neck BMD or fracture risk. Independent of treatment, femoral neck BMD decreased (*p* = 0.034), while risk of hip (*p* = 0.003) and major osteoporotic fracture (*p* = 0.044) increased. There was no mean difference in bone marker levels between groups from baseline to endpoint. These findings align with the lack of difference in BMD and fracture risk at the end of study.

**Conclusion:**

Probiotics did not alter BMD or fracture risk, as supported by bone cellular activity that was similar to the placebo group by the end of study.

**Supplementary Information:**

The online version contains supplementary material available at 10.1007/s11657-025-01589-2.

## Introduction

Emerging evidence suggests that probiotics support bone health outcomes through the modulation of the gut microbiome [1, 2]. Probiotics are defined by the World Health Organization as “live microorganisms which, when administered in adequate amounts, confer a health benefit on the host” [3]. Among the many microorganisms identified, the most commonly studied species are from the *Lactobacillus* and *Bifidobacteria* genera, both of which are naturally present within the gastrointestinal flora [4]. While probiotic effects in humans and rodents have been shown to be usually strain- and disease-specific [5–7], studies in rodent models support the possibility of using probiotics containing *Lactobacillus* bacteria to attenuate ovariectomy-induced bone loss and promote favorable bone structure at the femur, tibia, and/or spine [1, 2, 8–16]. A recent systematic review and meta-analysis reported several benefits of probiotics for bone health in ovariectomized rodent models [17]. Moreover, probiotic treatments modified the composition of the gut microbiome, reduced serum markers of inflammation, and suppressed osteoclast differentiation and/or activity, resulting in decreased bone resorption accompanying ovariectomy-related estrogen deficiency [1, 2, 8–11, 13].

In humans, few randomized control trials have investigated the effects of probiotics on bone health outcomes in postmenopausal women [18–20]. These trials provided *Lactobacilli* either as an isolated strain or a combination of strains. For example, in older women (*N* = 90; aged 75 to 80 years) with low bone mass (osteopenia), *L. reuteri* supplementation for 12 months resulted in greater bone mineral density (BMD) at the tibia compared to the control group using high resolution peripheral quantitative computed tomography (HR-pQCT) (primary outcome) with no effect on secondary outcomes including BMD measured by dual-energy X-ray absorptiometry (DXA) at the hip and spine [18]. Some bone protective effects were also demonstrated in a multicenter trial with postmenopausal women aged 45 to 70 years (*N* = 249). At baseline, 43% of participants were classified as having osteopenia [19]. Consumption of a multispecies probiotic (*L. paracasei* DSM 13434, *L. plantarum* DSM 15312, and *L. plantarum* DSM 15353; 1 × 10^10^ CFU) for 12 months prevented the loss of BMD at the lumbar vertebrae but not the hip [19]. The authors noted that the lack of difference at the hip may have been due to differential effects of the probiotic treatment on trabecular and cortical bone. The smaller region of trabecular bone at the hip, compared to the lumbar vertebrae, could have contributed to the differences in the findings between these bone sites. Using a different combination of species (*L. casei* 1.3 × 10^10^ CFU; *B. longum* 5 × 10^10^ CFU; *L. acidophilus* 1.5 × 10^10^ CFU; *L. rhamnosus* 3.5 × 10^9^ CFU; *L. bulgaricus* 2.5 × 10^8^ CFU; *B. breve* 1 × 10^10^ CFU; *Streptococcus thermophiles* 1.5 × 10^8^ CFU), no probiotic effect on BMD of the spine was demonstrated at 6 months in postmenopausal women with osteopenia (*N* = 50; age 50 to 72 years). However, the consumption of the probiotic treatment reduced serum markers of bone formation (bone-specific alkaline phosphatase, BALP), bone resorption (cross-linked C-telopeptide of type I collagen, CTx), and inflammation (tumor necrosis factor alpha) [20]. A longer study duration of 12 months may be required to observe beneficial changes to BMD [21].

The present study adds to this existing literature, to further understand the potential supportive role of different probiotic strains on bone health in postmenopausal women (age 40 to 59 years). The primary objective was to determine if probiotic consumption (*L. brevis* HA-112 and *L. paracasei* HA-274) could attenuate the loss of femoral neck BMD while also determining an effect on fracture risk and markers of bone cell activity as secondary outcomes. This study was designed a priori as an extension of a randomized, double-blind, placebo-controlled trial that investigated the effects of the probiotic supplement on menopausal symptoms (primary objective).

## Methods

The study was performed over 48 weeks and involved two data collection sites based in the USA. The study was prospectively registered on ClinicalTrials.gov (Identifier: NCT04001088, 06/25/2019) and was approved by the Advarra Institutional Review Board (CR00228042; CR00232642) and the Brock University Health Science Research Ethics Board (File #20–319). Conduct of the study assessments were in accordance with the International Conference on Harmonisation—Good Clinical Practice principles and applicable US Code of Federal Regulations.

### Study design

**Fig. 1:**
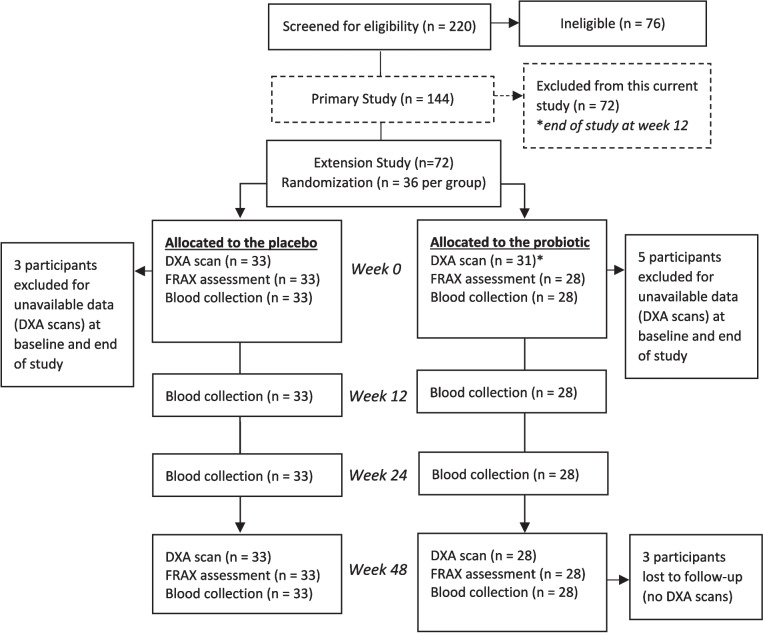
CONSORT flow diagram. *ITT analysis for BMD included 64 postmenopausal women (*n* = 33 women receiving the placebo; *n* = 31 women receiving the probiotic); PP analyses for BMD, FRAX, and serum BTMs included 61 postmenopausal women (*n* = 33 women receiving the placebo; *n* = 28 women receiving the probiotic)

Postmenopausal women (ages 40 to 59 years) who had their last menstrual period at least 12 months prior to screening were included in this study (*n* = 72). Eligible participants met all of the following inclusion criteria: vaginal pH of 5 or greater, Menopause Rating Scale score of 20 or greater [22], as well as willingness to provide written informed consent, consume the intervention allocated, complete all study assessments, and discontinue the consumption of supplements and foods containing added probiotics and/or prebiotics after a 4-week wash-out period. Information regarding the exclusion criteria can be found in Table SI1. Participants were given multiple codes in the event of unblinding due to a serious adverse event. This was a double-blind study in which participants and study personnel were unaware of the study product dispensed. The randomization list was generated by personnel not involved in the study conduct or analysis to ensure blinding was in place for all aspects of the study. Participants received either probiotic capsules containing 7.5 billion CFU of *L. brevis* HA-112 and *L. paracasei* HA-274 or placebo capsules (*n* = 36 per group). Investigational products were manufactured and provided by Lallemand Health Solutions (Mirabel, QC, Canada). Participants were instructed to keep the capsules refrigerated and to take one capsule with a meal at the same time every day for 48 weeks (Fig. 1). Self-administered daily diaries were collected and reviewed weekly to monitor compliance, concomitant medication(s), and/or supplements, as well as adverse events (Table SI2).

### Femoral neck BMD

DXA was used to measure femoral neck BMD in participants receiving either probiotic or placebo at baseline (week 0) and endpoint (week 48). For each group, the average percent change of BMD from baseline was reported.

### Risk of fracture using FRAX® tool

Using the FRAX® tool for the USA (Hispanic or Caucasian) (https://frax.shef.ac.uk/FRAX/tool.aspx?country=9 [23, 24]), femoral neck BMD and clinical risk factors were included in the calculation for each participant’s 10-year probability of risk of hip fracture (total score, %), and risk of major osteoporotic fracture (total score, %) was calculated. This tool has greater sensitivity for risk of fracture compared to BMD alone, as it is inclusive of clinical risk factors of fragility fracture including age, sex, height, weight, history of previous fracture, parent fractured hip, current smoking, glucocorticoids, rheumatoid arthritis, secondary osteoporosis, and alcohol consumption [23, 24]. Information about these clinical risk factors was collected via daily diaries.

### Serum bone turnover markers

Participants fasted for 12 h prior to blood collection at weeks 0, 12, 24, and 48. Concentrations of serum markers of bone formation, including procollagen type 1 N-terminal propeptide (P1NP) and BALP, were determined using commercially available enzyme-linked immunosorbent assay (ELISA) kits: Human P1NP, NBP2-76,465, Lot DQK2NACN89 (NovusBIO, Bio-techne), and Human BALP, MBS166762, Lot 202,110,001 (MyBioSource). Bone resorption activity was assessed by serum CTx using the Human CTx kit, NBP2-69,073, Lot 1UJ5QC2DQ9 (NovusBIO, Bio-techne), and bone turnover was evaluated using the Human Osteocalcin (OC) DuoSet, DY1419-05, Lot P284796 (R&D Systems, Bio-techne) along with DuoSet Ancillary Reagent Kit 3, DY009, Lot P296095 (R&D Systems, Bio-techne). All analyses were performed in triplicates and as per the manufacturers’ instructions.

### Statistical analyses

Sample size was based on a priori power analysis for the primary study and outcome (change in global Menopause Rating Scale score) rather than this extension study. Identifying bone density changes would require 120 subjects per group using the same methods used in the power analyses for the primary study and data from Jafarnejad et al. [20] and Kruger et al. [15] on serum CTX (power = 0.80 and alpha = 0.05) [Stata v14.4 statistical software; StataCorp, College Station, TX]. A per-protocol (PP) analysis was selected a priori for the bone health outcomes, as these were exploratory in nature, and the goal was to assess the efficacy of the intervention under ideal conditions. However, we acknowledge that an intention-to-treat (ITT) analysis is considered best practice. Accordingly, both ITT and PP analyses were conducted for the primary outcome, femoral neck BMD.

Overall, there was good compliance for both intervention groups in which all participants consumed at least 92% of the investigational product, with exception of three participants in the probiotic group that consumed less than 70% of the probiotic supplements. PP analyses included participants that consumed 80% or more of the intervention and completed a DXA scan at baseline. Sensitivity analyses, stratified by self-reported ethnicity (“Hispanic or Latino” or “not Hispanic or Latino”), were based on the ITT and PP analyses for femoral neck BMD. Secondary outcomes (FRAX and serum bone turnover markers, BTMs) were analyzed using PP analyses only. The three participants excluded in the PP analyses did not undergo a DXA test at the end of study or provide adequate blood samples throughout the study. Femoral neck BMD and FRAX scores (hip fracture and major osteoporotic fracture) were analyzed using a 2 (placebo, probiotic) by 2 (week 0, week 48) repeated measures linear model. Residuals of the models were screened such that assumptions were met, specifically normality, homogeneity of variance, and sphericity. Significant differences were followed-up by Tukey’s HSD post-hoc test. Serum BTMs were analyzed using a 2 (placebo, probiotic) by 3 (week 12, week 24, week 48) repeated measures linear model adjusted for baseline measure included as a covariate. Verification of assumptions and statistical analyses were conducted similarly to the procedures described above. Data for serum BTMs showed a non-normal distribution and were transformed using a natural log. All analyses were performed using the IBM SPSS Statistics v28.0.1.0 software and a significance of *p* < 0.05.

## Results

The participant flow diagram showed that of the 72 women that were included in this extension study, 64 women completed the 48-week intervention (*n* = 33 women receiving the placebo [age 53.4 ± 4.5 years (min–max = 42.1–60.0)]; *n* = 31 women receiving the probiotic [age 54.0 ± 3.9 years (min–max = 46.6–59.5)], Fig. 1). Eight participants were excluded due to unavailable DXA scans at baseline and end of study. Findings from Little’s test of missing completely at random determined that the missing data was completely at random (*p* = 0.935) and mean imputation was used for further analyses.

There were no significant differences in participants’ demographic characteristics between intervention groups at baseline (Table 1). Most participants self-identified as Hispanic or Latino ethnicity (85%), White race (100%), and reported some high school education (50%). According to average height and weight, participants in the placebo group were considered obese (BMI = 31.5) while participants in the probiotic group were considered overweight (BMI = 27.8). Of consideration is that BMI does not measure body fat and given the significant body composition changes that may occur during the menopause transition [25], BMI may not be an accurate outcome to assess obesity status among this population. Using the screening questionnaire, 79% of participants in the placebo group (*n* = 26) and 82% of participants in the probiotic group (*n* = 26) reported having a history of cardiovascular and/or endocrine conditions (Table 1). Overall compliance was 94.4% for probiotic consumption (318 ± 32 tablets) and 98.1% for placebo consumption (330 ± 6 tablets). No serious adverse events were reported.

**Table 1:** Demographics and characteristics of participants at baseline

	Placebo (*n* = 33)	Probiotic (*n* = 28)
Age (years)	53.5 ± 4.6	54.2 ± 3.9
40–44 (*n*)	1	0
45–49 (*n*)	6	6
50–54 (*n*)	12	10
55–60 (*n*)	14	12
Weight (kg)	79.7 ± 14.0	72.2 ± 10.7
Height (cm)	159.1 ± 8.0	161.6 ± 7.1
Hip (*T*-score)	− 0.93 ± 0.88	− 0.84 ± 1.07
Ethnicity (*n*)*		
Not Hispanic or Latino	6	3
Hispanic or Latino	27	25
Race (*n*)*		
White	33	28
Education (*n*)*		
No schooling	1	1
Some high school (no diploma)	14	17
High school graduate	8	6
College (no degree)	2	2
College	2	1
Bachelor’s degree	3	0
Master’s degree	1	0
Doctorate degree	1	0
Professional degree	1	1
Medical history (*n*)*		
Type 2 diabetes	11	13
Hypertension	14	10
Dyslipidemia	15	14
Hypothyroidism	6	8

Data are presented as mean ± SD for age, weight, height, and *T*-score, and counts of number of participants for ethnicity, education, and medical history

*Self-reported data; participants were instructed to select one of the ethnic categories (Hispanic or Latino, not Hispanic or Latino, Caucasian, not Caucasian, not reported, or unknown) as well as to select all of the applicable race categories (American Indian or Alaska Native, Asian, Black or African American, Native Hawaiian or Other Pacific Islander, White, other, not reported)

At baseline, femoral neck BMD did not significantly differ between groups. Sixty percent of women in the placebo group (*n* = 20) and 75% of women in the probiotic group (*n* = 21) had low bone mass (*T*-score between − 1.0 and − 2.5). One participant in the placebo group had osteoporosis (*T*-score − 2.5 or below), and all other participants had normal bone mass (*T*-score − 1.0 or above). At week 48, the distribution of participants with low bone mass increased to 69.7% in the placebo group (*n* = 23), and no change occurred in the probiotic group. 

### Primary outcome: ITT and PP analyses for femoral neck BMD

**Fig. 2:**
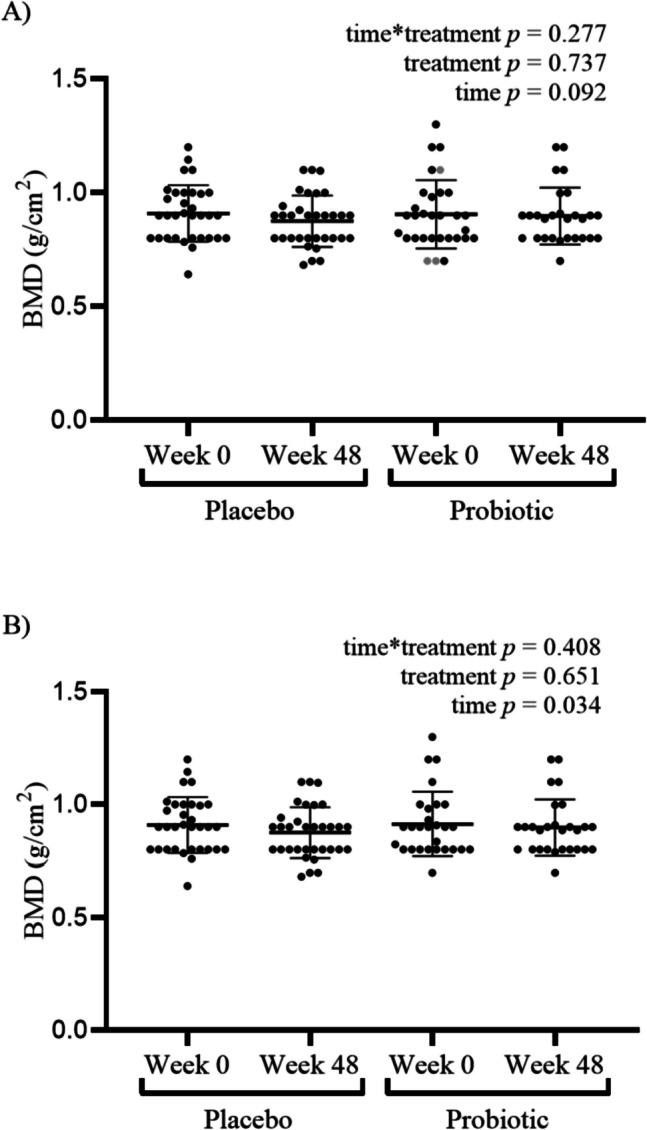
Femoral neck BMD: DXA results in women consuming the placebo or probiotic supplement at baseline (week 0) and end of study (week 48). **A** ITT: *n* = 33 women receiving the placebo, *n* = 31 women receiving the probiotic supplement (grey points represent women that were included in the ITT analysis only). **B** PP: *n* = 33 women receiving the placebo, *n* = 28 women receiving the probiotic supplement. Data are presented as mean ± SD. [GraphPad software version 9.5.1 for Windows, San Diego, CA, USA]

For both the ITT and PP analyses, there was no significant interaction between time and intervention on femoral neck BMD (ITT analysis, *p* = 0.277; PP analysis, *p* = 0.408; Fig. 2). Femoral neck BMD did not differ between intervention groups (ITT analysis, *p* = 737; PP analysis, *p* = 0.651). Regardless of intervention group, the effect of time was not demonstrated in the ITT analyses (mean difference =  − 0.021, 95% CI [− 0.045, 0.003], *p* = 0.092). However, PP analyses showed that BMD was lower at week 48 compared to baseline (*p* = 0.034).The mean relative change of femoral neck BMD over the study duration was − 1.75% in the probiotic group and − 3.74% in the placebo group.

### Sensitivity analysis

The results for the sensitivity analyses are presented in Table SI3. The demographic was skewed towards a significantly larger proportion of postmenopausal women who self-reported as “Hispanic or Latino” (PP analysis, *n* = 52; ITT analysis, *n* = 55) compared to postmenopausal women who self-reported as not “Hispanic or Latino” (PP analysis, *n* = 9; ITT analysis, *n* = 9). For both the ITT and PP analyses, probiotic supplementation had no effect on femoral neck BMD in postmenopausal women regardless of their self-reported ethnicity. Based on the PP analysis, femoral neck BMD decreased over time in "Hispanic or Latino" (p = 0.019), but no time effect was observed in "not Hispanic or Latino"(*p* = 0.310). Based on the ITT analyses, there was no effect of time on femoral neck BMD in “Hispanic or Latino” (*p* = 0.060) or “not Hispanic or Latino” (*p* = 0.310).

#### Secondary outcomes: FRAX scores and serum BTMs by PP analysis

There was no significant interaction between time and intervention on risk of hip fracture (*p* = 0.914) and risk of major osteoporotic fracture (*p* = 0.382; Table 2). Risk of hip fracture (*p* = 0.294) and risk of major osteoporotic fracture (*p* = 0.324) were not different between postmenopausal women consuming the probiotic and those who received the placebo (Table 2). In both groups, the risk of hip fracture (*p* = 0.003) and risk of osteoporotic fracture (*p* = 0.044) increased from baseline to endpoint (Table 2).

**Table 2:** Risk of fracture using FRAX® tool: PP analysis for FRAX scores estimating the 10-year probabilities of risk of major osteoporotic fracture and risk of hip fracture in postmenopausal women consuming the placebo or probiotic supplement at baseline (week 0) and the end of the study (week 48)

	Placebo (*n* = 33)	Probiotic (*n* = 28)
Major osteoporotic fracture, %		
Week 0	3.60 ± 1.87	3.35 ± 1.25
Week 48	4.21 ± 2.25	3.60 ± 1.24
Mean difference (W48–W0)	0.61 ± 1.61	0.25 ± 1.64
Visit*Treatment, *p*-value	0.382	
Treatment, *p*-value	0.324	
Visit,* p*-value	0.044	
Hip fracture, %		
Week 0	0.19 ± 0.16	0.24 ± 0.20
Week 48	0.24 ± 0.17	0.29 ± 0.26
Mean difference (W48–W0)	0.05 ± 0.11	0.05 ± 0.11
Visit*Treatment, *p*-value	0.914	
Treatment, *p*-value	0.294	
Visit, *p*-value	0.003	

Data are presented as mean ± SD

There was a significant interaction between time and treatment for serum P1NP (*p* = 0.003) and CTx (*p* = 0.006), but not serum BALP (*p* = 0.607) or OC (*p* = 0.442; Table 3; Fig SI1). At week 12, serum P1NP was lower in the probiotic group compared to the placebo group (mean difference [probiotic-placebo] =  − 0.217; *p* = 0.008) but not at week 24 (*p* = 0.217) or week 48 (*p* = 0.225). Within the probiotic group, there was an increase in serum P1NP at week 24 (mean difference [week 24–week 12] = 0.196; *p* = 0.006) that was maintained at week 48 (mean difference [week 48–week 12] = 0.176; *p* = 0.017). Within the placebo group, serum P1NP was lower at week 48 compared to week 24 (mean difference [week 48–week 24] =  − 0.186; *p* = 0.007). Serum CTx was more elevated in the probiotic group compared to the placebo group at week 48 (mean difference [probiotic-placebo] = 0.213; *p* = 0.042). There was no difference between intervention groups at week 12 (*p* = 0.376) or week 24 (*p* = 0.574). In the probiotic group, serum CTx was more elevated at week 48 compared to week 12 (mean difference [week 48–week 12] = 0.278; *p* < 0.001). No differences between time points were observed in the placebo group (*p* > 0.05). There was no main effect of treatment for serum BALP (*p* = 0.771) or OC (*p* = 0.637). There was a main effect of time in which serum BALP was more elevated at week 48 compared to week 12 (mean difference [week 48–week 12] = 0.186; *p* = 0.011), while serum OC was more elevated at week 24 compared to week 12 (mean difference [week 24–week 12] = 0.667; *p* = 0.008) but this change was not maintained by week 48 (mean difference [week 48–week 12] = 0.074; *p* = 0.989).

**Table 3:** Serum BTMs: PP analyses for measures of bone formation (P1NP, BALP) and bone resorption (CTx, OC) in postmenopausal women consuming the placebo or probiotic supplement at weeks 0, 12, 24, and 48

	Placebo (*n* = 33)	Probiotic (*n* = 28)
P1NP (ng/mL)		
Week 0	59.02 ± 26.51	61.02 ± 28.60
Week 12	61.09 ± 25.30 ^cd^*	47.83 ± 15.13^a^*
Week 24	62.11 ± 25.27 ^d^	58.92 ± 21.72 ^b^
Week 48	50.56 ± 12.00 ^c^	57.12 ± 18.85 ^b^
Mean difference (W48–W0)	− 8.47 ± 4.75	− 3.91 ± 5.15
Visit*Treatment, *p*-value	0.003	
Treatment, *p*-value	0.197	
Visit, *p*-value	0.237	
BALP (ng/mL)		
Week 0	1.33 ± 0.31	1.25 ± 0.31
Week 12	1.33 ± 0.35 ^a^	1.23 ± 0.33 ^a^
Week 24	1.36 ± 0.36 ^ab^	1.31 ± 0.67 ^ab^
Week 48	1.47 ± 0.36 ^b^	1.47 ± 0.67 ^b^
Mean difference (W48–W0)	0.13 ± 0.09	0.22 ± 0.09
Visit*Treatment, *p*-value	0.607	
Treatment, *p*-value	0.771	
Visit, *p*-value	0.007	
OC (ng/mL)		
Week 0	3.99 ± 2.21	3.83 ± 1.49
Week 12	4.35 ± 2.28 ^a^	3.68 ± 2.18 ^a^
Week 24	4.75 ± 2.50 ^b^	4.62 ± 2.38 ^b^
Week 48	4.14 ± 2.15 ^ab^	4.04 ± 2.24 ^ab^
Mean difference (W48–W0)	0.15 ± 0.39	0.21 ± 0.43
Visit*Treatment, *p*-value	0.442	
Treatment, *p*-value	0.637	
Visit, *p*-value	0.006	
CTx (ng/mL)		
Week 0	0.31 ± 0.15	0.30 ± 0.14
Week 12	0.33 ± 0.12	0.31 ± 0.13 ^a^
Week 24	0.33 ± 0.12	0.34 ± 0.15 ^a^
Week 48	0.33 ± 0.12 *	0.43 ± 0.25 ^b^*
Mean difference (W48–W0)	0.01 ± 0.03	0.13 ± 0.03
Visit*Treatment, *p*-value	0.006	
Treatment, *p*-value	0.444	
Visit, *p*-value	0.330	

Data are presented as mean ± SD

*BALP* bone-specific alkaline phosphatase, *CTx* cross-linked C-telopeptide of type I collagen, *OC* osteocalcin, *P1NP* procollagen type 1 N-terminal propeptide

*p < 0.05 for time × treatment interaction effects between the probiotic group and placebo group at a denoted time point

^a,b,c,d^*p* < 0.05 for time effects between weeks 0, 12, 24, or 24

## Discussion

Daily oral intake of the probiotic supplement for 48 weeks had no significant effect on femoral neck BMD, the risk of hip fracture, and the risk of major osteoporotic fracture. Regardless of the intervention, femoral neck BMD was lower at week 48 compared to baseline, and this loss was aligned with an increased risk of hip fracture and major osteoporotic fracture over time. There was no mean difference in bone marker levels between groups from baseline to endpoint. The lack of probiotic effect on BMD and fracture risk could be attributed to the fact that the study sample was inclusive of postmenopausal women with normal bone mass (average *T*-score =  − 0.90 ± 0.97). Additionally, it is acknowledged that there is rapid bone loss following menopause [26–28], making it challenging to measure an effect of an intervention during these rapid changes in bone cellular activity. Of note is that previous studies that support the use of probiotic supplementation on bone health have been conducted in postmenopausal women with low bone mass [18, 19]. The studies conducted in Sweden suggested not all skeletal sites may benefit from probiotic supplementation and that there were no differences in serum bone markers (NTx or BALP) [18, 19]. In postmenopausal women ages 75–80 years (average *T*-score = − 0.66 ± 0.83) consuming *L. reuteri* ATCCPTA 6475 (1 × 10^10^ CFU) for 12 months, there was no effect on BMD of the total hip, femur neck or spine assessed by DXA, compared to the control [18]. However, in this same study, tibia analysis using HR-pQCT demonstrated significant differences in BMD and lower reduction in trabecular bone volume fraction, favoring the probiotic intervention [18]. Imaging with HR-pQCT may yield different results due to greater sensitivity for detection of subtle changes in BMD [29]. This imaging technique can also provide insight on trabecular and cortical bone structure properties that contribute to bone strength.

The bone promoting effects of *L. reuteri* ATCCPTA 6475 (1 × 10^9^ CFU) from this previous study [18] aligns with findings from studies in ovariectomized rodents. *L. reuteri* ATCCPTA 6475 attenuated ovariectomized-induced trabecular bone changes [1]. Greater bone volume fraction and trabecular number along with lower trabecular separation at the femur and greater trabecular thickness at the vertebrae was observed compared to the ovariectomized control group [1]. A combination of *Lactobacillus* strains (*L. paracasei* DSM 13434, *L. plantarum* DSM 15312 DSM 15313) showed similar effects on trabecular bone structure when the probiotic intervention was provided 1.5 weeks after ovariectomy surgery and greater cortical thickness when the intervention was provided 2 weeks prior to ovariectomy surgery [11, 12]. This research group also demonstrated greater BMD at the lumbar spine following 12 months of consumption of the probiotic treatment (*L. paracasei* DSM 13434, *L. plantarum* DSM 15312 DSM 15313) in postmenopausal women age 45–75 years (*T*-score/bone mass) [19]. Interestingly, no differences were observed at the hip or femur neck or for serum bone turnover markers (P1NP, CTx, OC) between groups [19]. Understanding the response of systemic bone cellular changes to probiotics requires further investigation.

A strength of our study is the dominant representation of Hispanic postmenopausal women given the paucity of data for this population. While there is some evidence to support ethnic differences in BMD, rate of bone loss with aging and risk of fracture [30–33] results from studies investigating differences in mean BMD between Hispanic and non-Hispanic individuals have been inconsistent [34–37]. Eighty-five percent of participants in the current study self-identified as Hispanic or Latino. The vast majority of bone research evaluating BMD and risk of fracture in the USA has been conducted among non-Hispanic populations, and less is known about ethic and/or racial differences in bone health [30–39]. Based on the sensitivity check, the similar results between entire sample and when stratified by ethnicity (“Hispanic or Latino,” “not Hispanic or Latino”) suggest the findings are related to a Hispanic sample, despite inclusion of the small proportion of Caucasian women included in the sample population. With a combination of biological, environmental, and cultural factors that can influence the evolution of the gut microbiome [40], the strain-specific role of probiotics for modulating the gut microbiome may differ across ethnic groups.

Limitations of this study include sample size and a focus on a single skeletal site. The use of medications that can modulate bone health was recorded at screening and participants were instructed to continue reporting concomitant medication(s) in their daily diary throughout the study duration. No use of osteoporosis medications was reported, and this is likely because participants were not recruited based on BMD. Most of the participants in our sample (*n* = 57; 93%) reported active use of numerous concomitant medications to address cardiovascular and/or endocrine medical conditions reported at screening (Table 1; Table SI4). The anti-inflammatory effects of the medications reported may have obscured bone findings. It can be speculated that the long-term use of the combination of drugs that have been shown to have an effect on BMD and/or fracture risk, such as angiotensin-converting enzyme inhibitors [41–43] and statins [44], could mask potential probiotic effects due to their role in regulating inflammatory cytokines. Given this was a modest sample size and underpowered for detecting differences in bone health outcomes, this probiotic supplement should be studied in a future trial that includes BMD and/or fracture as a primary outcome. Moreover, a longer study duration, a higher dose, or assessment at multiple bone sites using HR-pQCT at the tibia or radius, as well as DXA measurement of other bone sites (i.e., lumbar spine and/or ultradistal radius), should be considered in the design of future studies. Furthermore, not all skeletal sites may experience a benefit of the probiotic supplement, and additional skeletal sites may need to be considered.

In summary, there was no effect of a novel combination of *L. brevis* HA-112 and *L. paracasei* HA-274 on BMD or fracture risk. Consideration of diverse populations is valuable for understanding potential mechanisms as well as the sociodemographic factors that may influence how we can tailor the use of probiotic treatments as a strategy to promote bone health.

## Supplementary Information

Below is the link to the electronic supplementary material.

Supplementary file1 (DOCX 95 KB)

## Data Availability

The data that supports the findings of this study are available on request from the Study Sponsor. The data is not publicly available due to privacy or ethical restrictions.
