# Supplementary material for: Using probiotic supplementation to support bone health in postmenopausal women: a randomized, double-blind, parallel, placebo-controlled, multi-center study
Source: Arch Osteoporos. 2025 Jul 27;20(1):103. doi: 10.1007/s11657-025-01589-2 (PMC12296836; doi:10.1007/s11657-025-01589-2)
Supplement: Supplementary file 1 — Supplementary file1 (DOCX 95 KB) [file 11657_2025_1589_MOESM1_ESM.docx]

Table SI1. Eligibility criteria.

| Inclusion criteria | - Post-menopausal woman aged 40 to 59 years - Last menstrual period has occurred at least 12 months prior to screening - Vaginal pH ≥ 5 - Menopause Rating Scale score ≥ 20 - Willing and able to give written informed consent. - Willing to consume IP or placebo, complete questionnaires, records, and daily diaries associated with the study and to complete all clinical trial visits. - Willingness to discontinue consumption of probiotic supplements and food containing added probiotics and/or prebiotics |
| --- | --- |
| Exclusion criteria | Health Status:   - History of cancer (except localized skin cancer without metastases) 5 years prior to screening. - History of any clinically significant disease or disorder which, in the opinion of the investigator, may either put the potential subject at risk because of participation in the study, or influences the results (*according to the primary outcome*) or the potential subject's ability to participate in the study. - History or presence of gastrointestinal, hepatic, or renal disease, or any other condition known to interfere with absorption, distribution, metabolism, or excretion of drugs - History of a surgical procedure for the treatment of obesity - Participants previously diagnosed with hyperparathyroidism and/or hyperthyroidism.   Lifestyle factors:   - Milk or soy allergy. - Use of any antibiotic drug within 1 month of screening. - History of alcohol or drug abuse in the 12 months prior to screening. - Currently consuming more than 2 standard alcoholic beverages a day.   Vaginal health/menopause:   - Induced menopause through full/partial hysterectomy, chemotherapy, or radiation. - Consumption of natural health products used to treat menopausal symptoms such as phytoestrogens, Black Cohosh, dehydroepiandrosterone, Dong Quai, vitamin E (>250 IU/day), high consumption of soy food products. - Currently undergoing or have previously undergone hormone therapy for treatment of menopausal symptoms. - Active vaginal infections/abnormalities. - Use of any vaginal medication, vaginal rinses and/or moisturizers, gels containing xylocaine or other analgesic products to decrease pain during intercourse, 1 week before and during study. |

Table SI2. Schedule of assessments.

| Procedures/assessments | Screening – Pre-Baseline | Randomization – Baseline (Week 0) | End of Primary Study (Week 12) | Start of Extension Study (Week 24) | End of Extension Study (Week 48) |
| --- | --- | --- | --- | --- | --- |
| Informed consent and demographic questionnaire | X |  |  |  |  |
| Review inclusion and exclusion criteria | X | X |  |  |  |
| Review concomitant medications | X | X | X | X | X |
| Review adverse events | X | X | X | X | X |
| Medical history and demographic questionnaire | X |  |  |  |  |
| Anthropometric measurements |  | X |  |  | X |
| Daily diary dispensed or TC reminder to complete | X | X | X | X |  |
| Daily diary returned for review |  | X | X | X | X |
| Randomization |  | X |  |  |  |
| Capsules dispensed with instructions |  | X | X | X |  |
| Capsules returned & compliance check by capsule count |  |  | X | X | X |
| DXA scan* |  | X |  |  | X |
| FRAX analysis* |  | X |  |  | X |
| Blood draw for exploratory markers* |  | X | X | X | X |

*Denotes bone health outcomes

|  |  |  | **P-value – PP** | | | **P-value – ITT** | | |
| --- | --- | --- | --- | --- | --- | --- | --- | --- |
|  | **Placebo** | **Probiotic** | **Time* treatment** | **Treatment** | **Placebo** | **Probiotic** | **Time* treatment** | **Treatment** |
| **Hispanic or Latino (n)** | | | | | | | | |
| W0 – PP (52) W0 – ITT (55) | 0.92 ± 0.11 0.92 ± 0.11 | 0.92 ± 0.15 0.91 ± 0.16 | 0.442 | 0.704 | **0.019** | 0.297 | 0.808 | 0.060 |
| W48 – PP (52) W48 – ITT (55) | 0.88 ± 0.10 0.88 ± 0.10 | 0.90 ± 0.13 0.90 ± 0.12 |  |  |  |  |  |  |
| Mean difference (W48 - W0) PP ITT | -0.04 ± 0.02  -0.04 ± 0.02 | -0.02 ± 0.02 -0.01 ± 0.02 |  |  |  |  |  |  |
| **Not Hispanic or Latino (n)** | | |  |  |  |  |  |  |
| W0 – PP (9) W0 – ITT (9) | 0.87 ± 0.18 0.87 ± 0.18 | 0.86 ± 0.06 0.86 ± 0.06 | 0.526 | 0.929 | 0.310 | 0.256 | 0.929 | 0.310 |
| W48 – PP (9) W48 – ITT (9) | 0.87 ± 0.16 0.87 ± 0.16 | 0.90 ± 0.10 0.90 ± 0.10 |  |  |  |  |  |  |
| Mean difference (W48 - W0) PP ITT | -0.002 ± 0.02 -0.002 ± 0.02 | 0.04 ± 0.03 0.04 ± 0.03 |  |  |  |  |  |  |

Table SI3. Sensitivity analyses based on PP and ITT for femoral neck BMD, g/cm^2^.

ITT, intent-to-treat analysis; PP, per-protocol analysis; W0, week 0 (baseline); W48, week 48 (end of study)

Table SI4. Concomitant medications.

|  | **Drug classification** | **Placebo (n)** | **Probiotic (n)** |
| --- | --- | --- | --- |
| **T2D** | Biguanides; Sulfonylureas | Metformin (10), Glimepiride (2), Glipizide (2) | Metformin (13), Glipizide (5), Glimepiride (1) |
| **Hypertension** | ACE inhibitor; angiotensin receptor blockers (ARBs); Beta-blockers; Calcium channel blcoker | Clonidnine (1), Lisinopril (13), Losartan (2), Enalapril (1), Atenolol (1), Carvedilol (1), Amlodipine (1) | Losartan (2), Lisinopril (8), Amlodipine (1), Olmesartin (1) Enalapril (1), Carvedilol (1) |
| **Dyslipidemia** | Statin (HMG-CoA reductase inhibitors) | Pravastatin (11), Atorvastatin (2), Simvastatin (3), Rosuvastatin (1) | Pravastatin (7), Atorvastatin (9) |
| **Hypothyroidism** | Hormones | Levothyroxine (8), Nature throid (1) | Levothyroxine (5), Nature throid (3) |
| **Asthma** | Bronchodialators; Leukotriene receptor antagonists | Montelukast (1) | Albuterol (1) |
| **Migraine, Epilepsy** | Anticonvulsants | Topiramate (1), Pregabalin (1), Frovatriptan (1) |  |
| **Appetite suppressant** | Anorectic | Phentermine (1) |  |
| **Bacterial infection** | Antibiotic | Amoxicillin (2) | Amoxicillin (1), Clindamycin (1) |
| **Allergies** | Antihistamine | Cetirizine (2), Hydroxyzine HCL (1), Pheniramine (1), Loratadine (1), Doxylamine succinate (1), Levocetirizine (1), Diphenhydramine (2), Promethazine (1) | Loratadine (3) |
| **Gastrointestinal, Heartburn, Nausea** | H2 receptor antagonist; Proton pump inhibitor; antimetics | Omeprazole (1), Cimetidine (1), Ondansetron (2) | Famotidine (1), Simethicone (1) |
| **Edema** | Diuretic | Lasix (1), Chlorthalidone (1), Hydrochlorothiazide (7), Hiazide (1) | Hydrochlorothiazide (8) |
| **Anxiety, Depression** | Selective serotonin-norepinephrine reuptake inhibitors (SSNRIs) | Duloxetine (2) | Citalopram (1), Bupropion HCl (1) |
| **Pain** | Analgesics | Oxycodone (1), Acetaminophen (3), Hydrocodone (1), Excedrin (1) | Acetaminophen (1), Excedrin (1) |
| **Muscle spasms** | Tricyclic antidepressant derivative; Anticholinergics; Alpha-2 Adrenergic Agonists | Oxybutynin (1), Tizanidine (1) | Cyclobenzaprine (2), Nortriptline (1), Guanfacine (1) |
| **Inflammation** | Nonsteroidal anti-inflammatory drugs (NSAIDs) | Ibprofen (2), Aspirin (2) | Ibprofen (2) |
| **Hormone therapy** | Hormones |  | Estradiol (1), Progesterone (1) |

*Note*: Counts of the number of participants are identified within the brackets following each medication.


**Fig SI1** Serum bone markers: a) procollagen type 1 N-terminal propeptide, P1NP; b) osteocalcin, OC; c) bone-specific alkaline phosphatase, BALP; and d) cross-linked C-telopeptide of type I collagen, CTx measures in postmenopausal women consuming the placebo (black bars; n = 33) or probiotic supplement (white bars; n = 28) at weeks 0, 12, 24 and 48. *Denotes a significant difference between intervention groups at the time point (time*treatment, *p* < 0.05). Data are presented as mean ± SD. [GraphPad software version 9.5.1 for Windows, San Diego, CA, USA]
